# A New Role for the GARP Complex in MicroRNA-Mediated Gene Regulation

**DOI:** 10.1371/journal.pgen.1003961

**Published:** 2013-11-07

**Authors:** Alejandro Vasquez-Rifo, Gabriel D. Bossé, Evelyne L. Rondeau, Guillaume Jannot, Alexandra Dallaire, Martin J. Simard

**Affiliations:** Laval University Cancer Research Center, Hôtel-Dieu de Québec (Oncology-Centre Hospitalier Universitaire de Québec), Québec City, Québec, Canada; University of California San Diego, United States of America

## Abstract

Many core components of the microRNA pathway have been elucidated and knowledge of their mechanisms of action actively progresses. In contrast, factors with modulatory roles on the pathway are just starting to become known and understood. Using a genetic screen in *Caenorhabditis elegans*, we identify a component of the GARP (Golgi Associated Retrograde Protein) complex, *vps-52*, as a novel genetic interactor of the microRNA pathway. The loss of *vps-52* in distinct sensitized genetic backgrounds induces the enhancement of defective microRNA-mediated gene silencing. It synergizes with the core microRNA components, *alg-1* Argonaute and *ain-1* (GW182), in enhancing seam cell defects and exacerbates the gene silencing defects of the *let-7* family and *lsy-6* microRNAs in the regulation of seam cell, vulva and ASEL neuron development. Underpinning the observed genetic interactions, we found that VPS-52 impinges on the abundance of the GW182 proteins as well as the levels of microRNAs including the *let-7* family. Altogether, we demonstrate that GARP complex fulfills a positive modulatory role on microRNA function and postulate that acting through GARP, *vps-52* participates in a membrane-related process of the microRNA pathway.

## Author Summary

The microRNA pathway is a post-transcriptional gene regulatory system that uses small non-coding RNAs called microRNAs to control multiple developmental and physiological processes. With the goal of unveiling factors modulating this regulatory pathway, we have undertaken the exploration of genetic interactors in the roundworm *Caenorhabditis elegans*. We identify *vps-52*, a component of the Golgi Associated Retrograde Protein or GARP complex, and establish that this complex executes a positive modulatory role on microRNA activity. The absence of *vps-52* function exacerbates diverse microRNA-related defects. Molecularly, this effect relates to decreased abundance of microRNAs and the GW182 proteins. Considering that GARP is involved in the traffic of vesicles from endosomes back to the Golgi apparatus, we propose that GARP facilitates a membrane-related process of the microRNA pathway.

## Introduction

The microRNA (miRNA) pathway is a gene regulatory system that uses small non-coding RNAs to target messenger RNAs (mRNAs) for post-transcriptional regulation. In the canonical form of miRNA biogenesis, miRNA-containing transcripts are processed through sequential cleavage operated by the Drosha and Dicer enzymes into mature miRNA species (21–23 nucleotides long) that associate with an Argonaute protein (reviewed in [1]). In its effector phase, the miRNA-loaded Argonaute, as part of the core miRNA-induced silencing complex (miRISC), regulates target mRNAs through binding sites in their 3′UTRs. The most detailed repressive effector function of this complex is mediated by its association to GW182 proteins (reviewed in [2]). The miRISC-mediated effector phase of target regulation, may involve the repression of multiple target molecules by each single miRNA through the process of ‘miRNA recycling’, and this mRNA regulation affects miRNA stability [3]–[7]. Finally, all miRISC components would be subjected to degradation, nucleases have been shown to degrade miRNAs (reviewed in [8]), and autophagy mediates the degradation of Dicer, Argonaute [9], [10] and GW182 [11].

In the nematode *Caenorhabditis elegans (C. elegans)*, the miRNA pathway comprises over 120 miRNAs [12], two GW182 homologues (*ain-1* and *ain-2*) [13], [14], the Argonautes *alg-1* and *alg-2* (both referred to as *alg-1/2*) [15] and single genes for Dicer (*dcr-1*) [15], [16] and Drosha (*drsh-1*) [17]. In worms, as in other animals, the miRNA pathway is essential for development and reproduction. Animals mutant for *dcr-1* or *drsh-1* genes are sterile [15]–[17], while at the Argonaute level, the loss of both *alg-1/2* results in embryonic arrest [15], [18]. In contrast, single mutants of *alg-1* or *alg-2* display differentially penetrant post-embryonic, somatic and germ line defects [19], [20]. As exemplified here, the existence of these two gene paralogs, with specialized and partially redundant functions provides an opportunity to study the miRNA pathway in a sensitized genetic condition where miRNA activity is reduced albeit not completely abolished, by screening for genetic enhancers of the partial loss-of-miRNA condition.

In the present study, we identify the *vps-52* gene, encoding a component of the GARP complex, as a genetic interactor of the miRNA-specific *alg-1* Argonaute, and establish that this complex fulfills a positive modulatory role in regulating the activity of miRNAs. The loss of *vps-52* in distinct sensitized genetic backgrounds induces the reiteration of the miRNA-controlled proliferative seam cell division program, enhances the *let-7*-related lethal phenotype, exacerbates the abnormal vulval development associated to the lessened miRNA-regulation of the *let-60* gene, as well as augments the defective expression of a reporter of the *lsy-6* miRNA activity in the ASEL neuron. Our phenotypic analyses thus suggest a broad role for GARP in miRNA function. Underpinning these GARP effects, we found decreased abundance of miRNAs and the GW182 proteins. Based on our data, we propose that the GARP complex operation facilitates a transition of miRISC occurring at endomembranes.

## Results

### The gene *vps-52* is a genetic interactor of the microRNA pathway Argonaute *alg-1*

**Figure 1:**
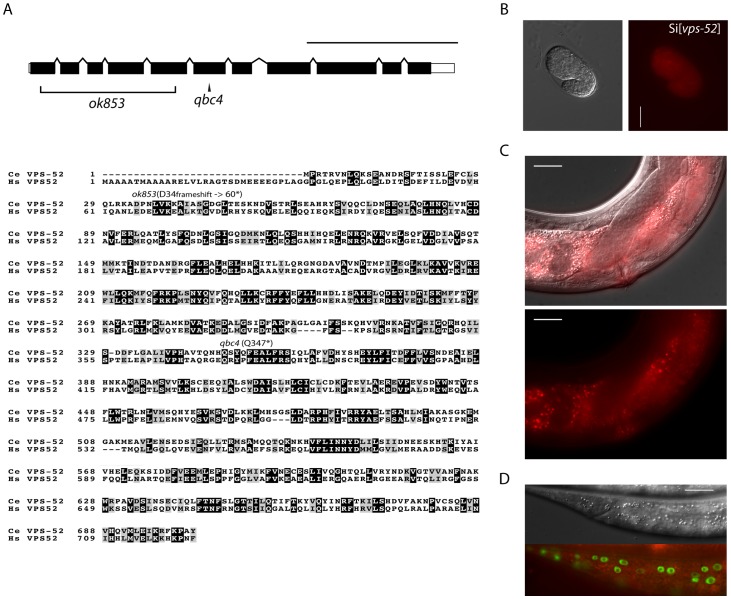
Features and mutants of the *vps-52* gene. **A**) (Top) Schematic representation of the intron-exon structure of the *C. elegans vps-52* gene. The two studied alleles are indicated (the allele *qbc4* is a non-sense mutation and the allele *ok853* is a frameshift deletion). VPS-52 contains predicted coiled coils stretches, but no distinct protein domains have been identified. (Bottom) *vps-52* is conserved from worms to humans. ClustalW alignment (edited with Boxshade program) of human (*Hs*) and *C. elegans* (*Ce*) VPS-52 orthologs, the proteins share a 56% of sequence similarity. The amino acid changes introduced by the two alleles are indicated. **B–D**) Expression of a single copy insertion of *Pvps-52*::*vps-52::mCherry::vps-52* 3′UTR (referred to as Si[*vps-52*]). **B**) The expression of VPS-52 in early embryos. Micrographs of Nomarski (left panel) and mCherry fluorescence (right panel). Scale bar measures 20 µm. **C–D**) The *vps-52* gene is widely expressed in somatic tissues. **C**) VPS-52 expression in the vulva and somatic gonad. Top panel: micrograph of merged Nomarski with mCherry fluorescence pictures. Bottom panel: micrograph of mCherry fluorescence. The VPS-52 protein localizes to the cytoplasm within punctated intracellular foci. Scale bar measures 20 µm. **D**) Expression of VPS-52::mCherry in the hypodermal cells. Micrographs of Nomarski (top) and merged GFP and mCherry fluorescence (bottom). Tagged VPS-52 (in red) is expressed in the cytoplasm around the seam cells nuclei (in green, GFP tagged). Scale bar measures 20 µm.

**Figure 2:**
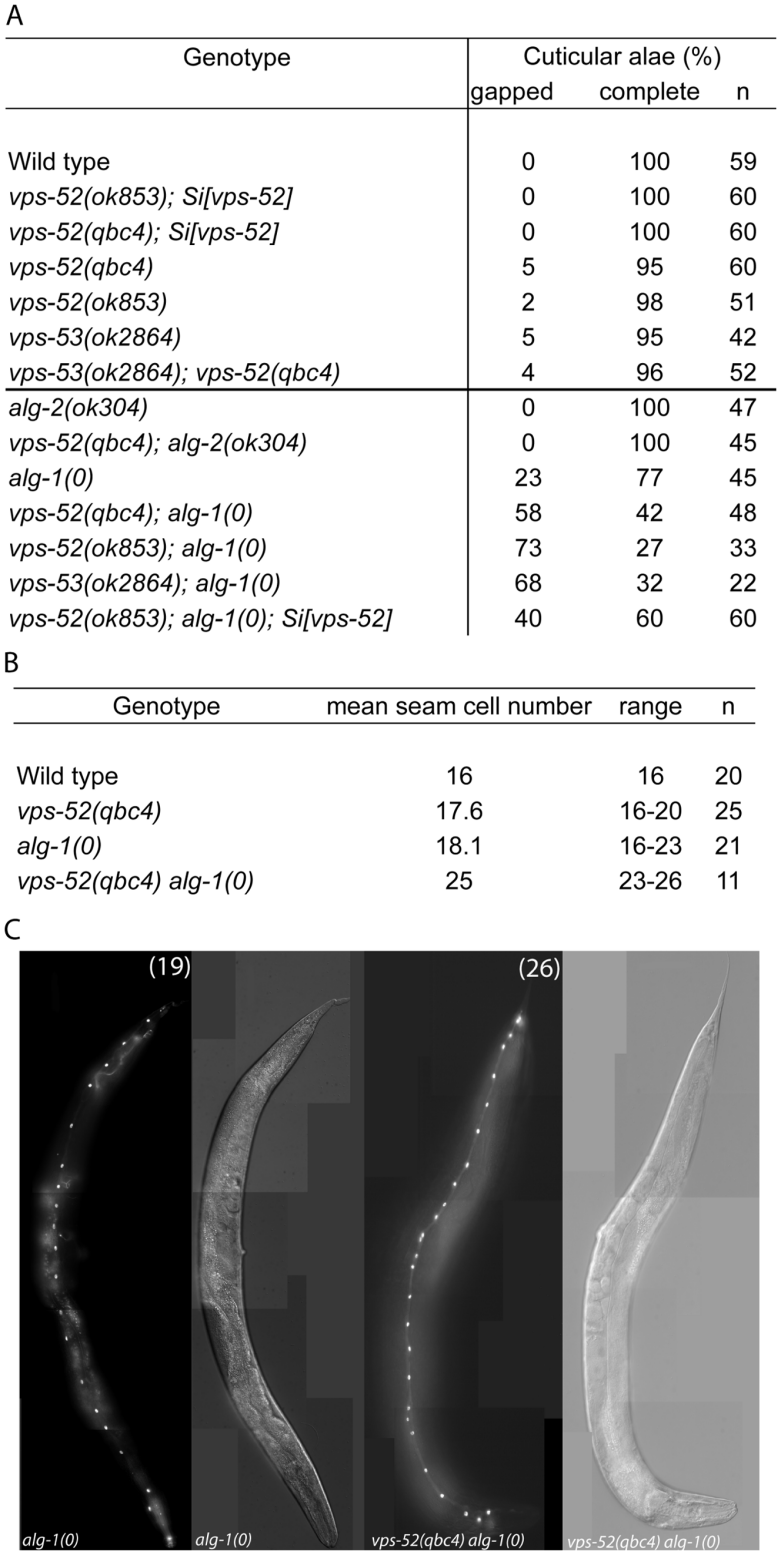
Seam cell-related phenotypes of the GARP mutants. **A**) Percentage of young adult worms with defective cuticular alae. Young adult hermaphrodites from synchronized populations were mounted and analyzed under Nomarski optics. The continuity of the cuticular alae was scored and categorized as gapped (one or more interruptions) or complete (no gaps present). Strains above the horizontal black line were scored at 20°C, those below that line were grown and scored at 15°C. **B**) Average and range of seam cells number at adulthood. The indicated strains were crossed with a strain expressing GFP in the seam cells (*scm::GFP*) and the number of seam cells scored in young adult hermaphrodites grown at 15°C. The mean value and range covered by the individual counts are indicated. **C**) Representative average of the seam cells at adulthood. The indicated mutants were crossed with a strain expressing GFP in the seam cells (*scm::GFP*) and scored. The pictures correspond to GFP and DIC fluorescence, the number of seam cell nuclei is indicated within parentheses. The number of animals scored (n) is indicated (**A**–**B**).

In order to identify new components and modulators of the miRNA pathway, we conducted a forward genetic screen for interactors of the *alg-1/2* Argonautes, based on a design that allows the recovery of gene enhancers, including synthetic lethal gene pairs [21]. In brief, we subjected to mutagenesis worms carrying a partially inheritable extrachromosomal array containing a functional GFP-tagged *alg-2* gene expressed in the *alg-2(ok304)* mutant (referred to as *alg-2* mutant) background. F2 clones were scored and selected as candidate interactors if their progeny was uniformly transgenic (*i.e.* there was no segregation of viable worms lacking the extrachromosomal array in the population), indicative of a possible genetic interaction of an unknown mutated factor, in the transgenic setting, with the *alg-2(ok304)* background. Upon removal of the screening background (array and *alg-2* mutation), a strain with increased growth and fertility defects in *alg-1/2*(RNAi) was selected, mapped and mutations identified by whole-genome sequencing (further details in the Materials and Methods section). We transgenically rescued the growth and fertility defects of the mutant strain, confirming the identity of the genetic interactor as *vps-52*. In addition to the single allele *vps-52*(*qbc4*) retrieved from the screen (Figure 1A), an available deletion allele, *vps-52(ok853)* was studied and found to display similar phenotypes (Figure 2). As expected, *vps-52* behaved genetically as an enhancer, but interestingly double mutants of *vps-52* with either *alg-2* or *alg-1* were obtained as viable strains, and the loss of *vps-52* induced a visible phenotypic enhancement in combination with the loss-of-function *alg-1(gk214)* mutant (referred to as *alg-1(0)*), but not with *alg-2(ok304)* as reported below. The mutant strain initially isolated from the screen does not sustain the segregation of viable animals without the extrachromosomal array, which could be accounted for by array-mediated overexpression effects of the GFP::ALG-2 fusion protein or additional secondary background mutations; its segregation was not further investigated.

The *vps-52* gene encodes a conserved structural protein (Figure 1A), that functions in the traffic of vesicles to the *trans*-Golgi network (TGN). It forms part of a conserved TGN-localized multimeric complex, known as the GARP (Golgi Associated Retrograde Protein) complex [22], [23], which comprises in *C. elegans* the genes *vps-51*, *vps-52*, *vps-53* and *vps-54* [24]. Expressing a functional fluorescently tagged VPS-52 from a single copy genomic insert controlled by endogenous gene regulatory elements (referred to as *Si[vps-52]*), we detected widespread VPS-52 expression in cytoplasmic puncta of many somatic tissues from early embryos on (Figure 1B–D), consistent with a previous report [24]. In particular, VPS-52 is expressed in temporal continuity at all larval stages in the vulval and seam cells (Figure S1).

### GARP mutants enhance the seam cell defects of the animals lacking the Argonaute *alg-1*

To address whether the phenotypes of *vps-52* mutants reflect an impairment of the GARP complex activity, we included in our study a strain defective for another subunit of this complex, the *vps-53(ok2864)* mutant. To analyze the function of *vps-52* and *vps-53* in the miRNA pathway, we first studied the development of seam cells. These lateral rows of hypodermal cells have a postembryonic developmental program, consisting of patterned rounds of division during each larval stage (L1 to L4), ended by terminal differentiation encompassing exit from the cell cycle, cell fusion and production of a cuticular structure (named alae) at the transition to adult. The seam cell developmental program is controlled at different larval stages by the miRNA lin-4 [25] and those of the let-7 family (miR-48, miR-84, miR-241 and let-7) [26], [27] and their targets *lin-14*, *lin-28*, *hbl-1*, *daf-12* and *lin-41* [26]–[33]. The repetition of the symmetrical seam cell division program that normally occurs once at the L2 stage is a frequently observed defect in mutants of core components of the miRNA pathway, such as *alg-1/2*, *dcr-1* and *ain-1*/2 [13], [15]. Similarly to these mutants, other pathway modulators and components also display distinctive seam cells defects [34]–[36]. Discontinuities in the cuticular alae (in particular, gaps) arise from inappropriate terminal differentiation, and are an indicator of possible alterations in the development of seam cells. We analyzed the *vps-52* and *vps-53* mutants and noticed mild penetrant defects on the alae structures (Figure 2A) that were not exacerbated in the *vps-52*; *vps-53* double mutant, demonstrating epistasis consistent with the affiliation of both gene products to a common complex.

We then proceeded to analyze the defects of the *vps-52* and *vps-53* mutants (referred to as GARP mutants) in the absence of functional *alg-1/2* Argonautes. Given that defects of the *alg-1* null mutant are less penetrant at lower temperatures (data not shown), we conducted the following scoring of alae and seam cell counts under more beneficial condition (15°C) to allow for better phenotypic enhancement. No enhancement of alae defects was observed in *vps-52(qbc4)*; *alg-2* double mutant animals (Figure 2A). However, combining either *vps-52* or *vps-53* with the *alg-1* null mutant caused a very prominent increase in alae defects, which were partially rescued by transgenic *vps-52* expression (Figure 2A). The enhancement of the *alg-1(0)* alae defects was correlated with an increase in the number of seam cells. While single GARP mutants showed minor deviations from the wild-type lineage (16 seam cells at adulthood), the mean number of seam cells in *alg-1* mutant at 15°C (18 cells), reached 25 in *vps-52(qbc4) alg-1(0)* (Figure 2B–C). This increase in seam cells was not observed in the L1 larval stage (data not shown), indicating that it likely results from the reiteration of the L2 stage proliferative division of the seam cells. We then addressed whether the effects of *vps-52* mutant on the seam cell phenotype of *alg-1(0)* worms were recapitulated with related mutants of vesicle trafficking processes. We tested the effect of disrupted Golgi trafficking by impairing the action of the worm small GTPase *rab-6.2*, whose gene product physically interacts with the GARP complex [24]. While single mutants of the putative null *rab-6.2(ok2254)* did not display any gapped alae, the exposure of *rab-6.2(ok2254)* to *alg-1(RNAi)* caused a strong interruption in the continuity of alae (Table S1). Moreover, this disruption was much stronger than that obtained for *vps-52(qbc4)* assayed under the same experimental conditions (100% vs 41% gapped alae; Table S1). We conclude that the loss of *vps-52* or *vps-53* function does not prominently affect seam cell development, but effectively synergizes in the absence of ALG-1 to induce reiteration of seam cell division program. Our observations of similar synergy for the mutant of *rab-6.2*, implicate the Golgi-associated function of these genes and miRNA-controlled development of the seam cells.

### The loss of GARP enhances the defects of miRNA mutants in a target-dependent manner

The let-7 family members miR-48, miR-84 and miR-241 redundantly regulate the expression of their target gene *hbl-1* during the L2-to-L3 larval stage transition [26], [28]. Abolishing completely the function of these three miRNAs causes seam cells to reiterate their L2 developmental program [26]. Loss of singleton or pairs of these genes leads to seam cell defects of varied penetrance, thereby constituting useful sensitized genetic backgrounds where the action of pathway modulators can be unveiled, as previously exemplified for the *nhl-2* modulator [36]. It was therefore possible that the altered seam cell development observed in *alg-1* mutant and enhanced by loss of *vps-52* resulted from impaired miR-48, miR-84 and miR-241 miRNAs function. Consequently, we evaluated whether GARP mutants would alter seam cell development in the absence of miR-48, with the *mir-48(n4097)* mutant allele (referred to as *mir-48(0)*). Although loss of this miRNA did not induce seam cells defects on its own, the concomitant loss of *vps-52* did lead to increase gapped or absent alae (Table 1). We next investigated the genetic interaction of *vps-52* with *hbl-1*, a main target of the *let-7* miRNA family that controls developmental timing at the L2 stage. Reduced *hbl-1* function results in the precocious terminal differentiation of the seam cells at the third larval molt evidenced by the production of alae [28], [36]. The combination of the reduced-function allele, *hbl-1(ve18)*, with a *vps-52* mutant resulted in the partial suppression of the *hbl-1(ve18)* precocious phenotype (Table 1 and Figure S2). Similarly, suppression of this *hbl-1* mutant phenotype has been reported upon concomitant loss of the let-7 miRNA family [36]. Altogether, these results suggest that loss of *vps-52* diminishes the activity of let-7 family miRNAs. This effect likely underpins the enhanced defects in the developmental program of the seam cells observed with the *alg-1(0)* and *mir-48(0)* mutants.

**Table 1:** Alae defects for the *vps-52*, *hbl-1* and *mir-48* mutant animals.

Genotype	Early L4 alae synthesis (%)			Adult alae (%)			
	no alae	alae	n	no alae	gapped	complete	n
Wild type	100	0	20	0	0	100	20
*vps-52(qbc4)*	100	0	20	0	5	95	60
*mir-48(0)*	*-*	*-*	*-*	0	0	100	20
*vps-52(qbc4); mir-48(0)*	*-*	*-*	*-*	4	22	74	23
*hbl-1(ve18)*	10	90	20	-	-	-	-
*hbl-1(ve18) vps-52(qbc4)*	45	55	20	-	-	-	-

The indicated strains were grown at 20°C and scored under Nomarski optics for alae synthesis and defects (absent, gapped or complete) at early L4 and young adult stages. The percentage of animals with absent, gapped or complete alae is indicated. The number of animals scored (n) is indicated.

We further extended our analysis to the let-7 miRNA, which regulates developmental programs at the L4 to adult transition. The complete loss of the *let-7* miRNA gene produces a highly penetrant phenotype of bursting through the vulva [27] that is classically used in phenotypic assessments of miRNA functions (*e.g.* [37]). In this respect, we did not observe any bursting phenotype in the GARP mutants (Table 2). In addition, the bursting of GARP mutants in combination with *alg-1(0)* was not overtly different from that of single *alg-1* mutant (Table 2). We then investigated if there was any effect on sensitized *let-7* genetic backgrounds. We used, to that aim, the hypomorphic *let-7* mutation, the *n2853* allele, that carries a point mutation in the miRNA seed region, which leads to reduced mature let-7 miRNA level [27], [38] and to temperature-sensitive reduction in the activity of this miRNA [27]. Remarkably, the GARP mutants strongly enhanced the *let-7(n2853)* bursting phenotype at permissive temperature (Table 2). The penetrance of the defect prevented the propagation of double *vps-52(qbc4) let7(n2853)* mutants to useful populations, but, upon combination with the transgenic *vps-52* gene, a rescued viable strain could be obtained (Table 2). The let-7 miRNA represses expression of *lin-41*, one of its major target genes; and reducing *lin-41* function suppresses the bursting phenotype of *let-7* mutants [27], [32], [39]. We therefore decided to verify whether the *lin-41* impairment could suppress the penetrant bursting of the double *vps-52(qbc4) let-7(n2853)* mutant. The combination of the *lin-41(ma104)* hypomorph with *vps-52(qbc4) let-7(n2853)* resulted in a viable triple mutant and the complete loss of bursting (Table 2). Thus, the effect of *vps-52* on the sensitized *let-7* background is dependent on its target, *lin-41*.

**Table 2:** Lethal vulva bursting phenotype observed in different mutant backgrounds.

Genotype	Lethality (% of bursters)	n	Temp
Wild type	0	60	20°C
*alg-1(0)*	3	60	“
*vps-53(ok2864)*	0	60	“
*vps-52(qbc4)*	0	60	“
*vps-52(ok853)*	0	60	“
*vps-52(ok853) alg-1(0)*	7	60	“
*let-7(n2853)*	29	42	15°C
*let-7(n2853); vps-53(ok2864)*	75	20	“
*let-7(n2853); vps-52(qbc4)*	100	20	“
*let-7(n2853); vps-52(qbc4); Si[vps-52]*	46	41	“
*let-7(n2853); vps-52(qbc4); lin-41(ma104)*	0	31	“
*let-7(n2853); control(RNAi)*	21	47	“
*let-7(n2853); alg-1(RNAi)*	82	33	“

The indicated strains (grown at 15°C or 20°C, as indicated) were scored for bursting through the vulva at the developmental transition to the adult stage. The percentage of bursted animals is shown. The number of animals scored (n) is indicated. The value for *let-7(n2853) vps-52(qbc4)* was determined from individually genotyped progeny of *let-7(n2853) vps-52(qbc4)/+* mothers. The same procedure was used for *let-7(n2853); vps-53(ok2864)* mutant. Animals were fed bacteria expressing either control (*control(RNAi)*) or *alg-1* targeting (*alg-1(RNAi)*) dsRNA, as indicated.

**Figure 3:**
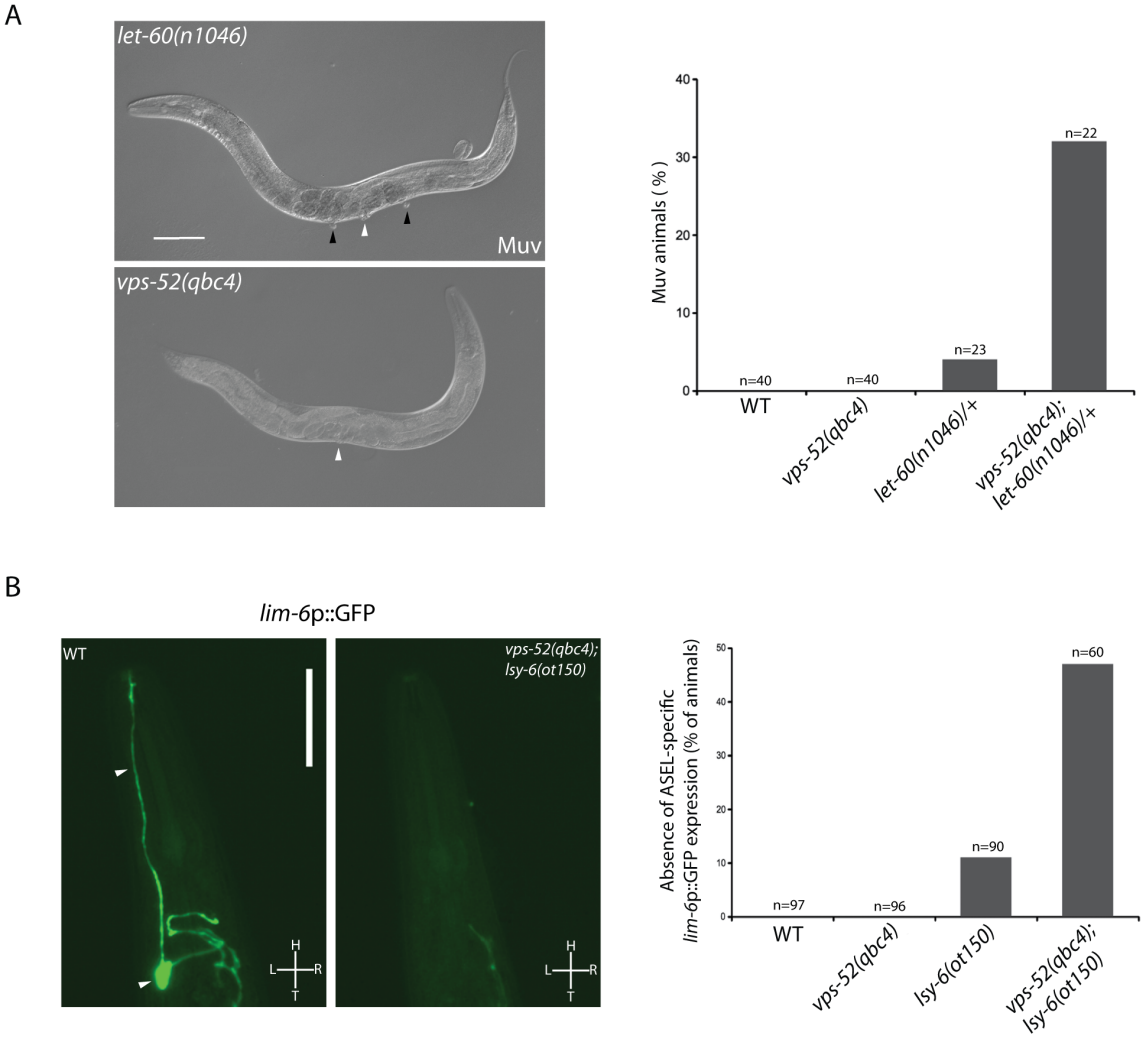
Effects of *vps-52* on the miRNA-mediated regulation of vulva and ASEL neuron development. **A**) Analysis of the *let-60*-mediated Muv phenotype. Left panels: Nomarski pictures of adult hermaphrodites of the indicated genotypes. A single vulva (white arrowhead) is observed in the GARP mutant. In *let-60(n1046)* mutants multiple vulval-like structures (black arrowheads, Muv phenotype) are observed in addition to vulva (white arrowhead). Scale bar measures 100 µm. Right panel: Percentage of adult worms of the indicated genotypes with the Muv phenotype. **B**) Analysis of the *lsy-6*-mediated ASEL-specific gene expression. Left Panels: GFP fluorescence pictures of the *lim-6*p::GFP reporter. In wild type (WT) animals, gene silencing mediated by the *lsy-6* miRNA leads to GFP expression in the ASEL neuron (arrowheads). Impairment of the miRNA activity in *vps-52(qbc4)*; *lsy-6(ot150)* mutant blocks reporter expression. Scale bar measures 50 µm. L: Left, R: Right, H: Head, T: Tail. Right panel: Percentage of adult worms of the indicated genotypes that failed to express the *lim-6*p::GFP reporter in ASEL. The strains were grown and scored at 15°C. The number of animals scored (n) is indicated.

We next expanded our study to an additional target of the let-7 miRNA family through the investigation of the regulation of vulva development by the miRNA-targeted *let-60* gene. During vulva development, an inductive signal from the somatic gonad promotes the vulval cell fates among a set of vulva precursor cells (VPCs). A restricted subset of VPCs adopts vulval fates and produces the egg-laying organ, while the remaining VPCs acquire a non-vulval epidermal fate [40]. The response to the inductive gonadal signal in the VPCs, requires the action of the *let-60* gene (RAS homolog). Increased *let-60* activity results in ectopic adoption of the vulval fate by VPCs and supernumerary vulva-like structures (known as the Muv phenotype) [41], [42]. The *let-7* family miRNAs participate in this developmental process through the regulation of their target gene *let-60* [36], [43]. The miRNA-mediated regulation of this gene can modulate the penetrance of the Muv phenotype in *let-60* gain-of-function mutants [36]. Similar to wild type worms, GARP mutants did not display the Muv phenotype (Figure 3A). A missense gain-of-function mutation, *let-60(n1046)* produces a weakly penetrant Muv phenotype in heterozygous condition (Figure 3A). The concomitant impairment of *vps-52* in *let-60(n1046)/+* mutants exacerbated the percentage of defective Muv animals (4% vs 32%; Figure 3A). This result suggests that *vps-52* gene function contributes to the negative regulation of *let-60* activity during vulva development, likely through the regulation of the *let-7* family miRNAs.

In order to address if GARP fulfills a restricted or broad modulatory activity on miRNA function, we then studied the regulation of ASEL neuron development by the *lsy-6* miRNA. The *lsy-6* miRNA promotes the adoption of a unique cell fate by the ASEL chemosensory neuron. This results in a functional right-left asymmetry between it and its counterpart, the ASER neuron [44]. In the ASEL neuron, the reduced expression of the *lsy-6* targeted gene *cog-1* leads to a subsequent gene regulatory cascade that activates ASEL-specific gene expression. In particular, the *lsy-6*-mediated silencing of *cog-1* expression leads to transcriptional derepression of *lim-6* [44]. Thus, a transcriptional fluorescent reporter of *lim-6* (*lim-6*p::GFP) serves as an indicator of achieved *lsy-6*-mediated *cog-1* silencing, when its expression is switched on in ASEL [44]. In wild type and *vps-52(qbc4)* animals, this reporter was expressed in the ASEL neuron of every worm (Figure 3B). In contrast, complete absence of *lsy-6* activity causes the total loss of expression of the reporter from the ASEL neuron [44]. While the hypomorphic *lsy-6(ot150)* mutation induced a mild penetrant loss of expression of this reporter (Figure 3B) [45], the loss of *vps-52* in *lsy-6(ot150)* mutants augmented the absence of reporter expression in ASEL (11% vs 47%; Figure 3B). This result suggests that *vps-52* gene function facilitates the activity of the *lsy-6* miRNA in silencing *cog-1* expression that subsequently contributes to the establishment of ASEL-specific gene expression.

In summary, our phenotypic analysis indicates that *vps-52* and *vps-53* mutants display weakly penetrant miRNA-related defects, but synergize with the *alg-1* and miRNA mutants in enhancing developmental defects in a miRNA target-dependent manner. In addition, the loss of *vps-52* suppresses the precocious phenotypes of a reduced function *hbl-1* mutant, increases the misregulation of the miRNA-targeted gene *let-60* during vulva development, as well as enhances the defective gene silencing activity of an hypomorphic *lsy-6* mutant in the ASEL neuron. These collective results, obtained in the context of distinct miRNA-dependent phenotypes in sensitized genetic backgrounds, suggest therefore, a positive and general role for the GARP complex in miRNA function.

### The loss of GARP leads to reduced abundance of the GW182 protein and miRNAs

**Figure 4:**
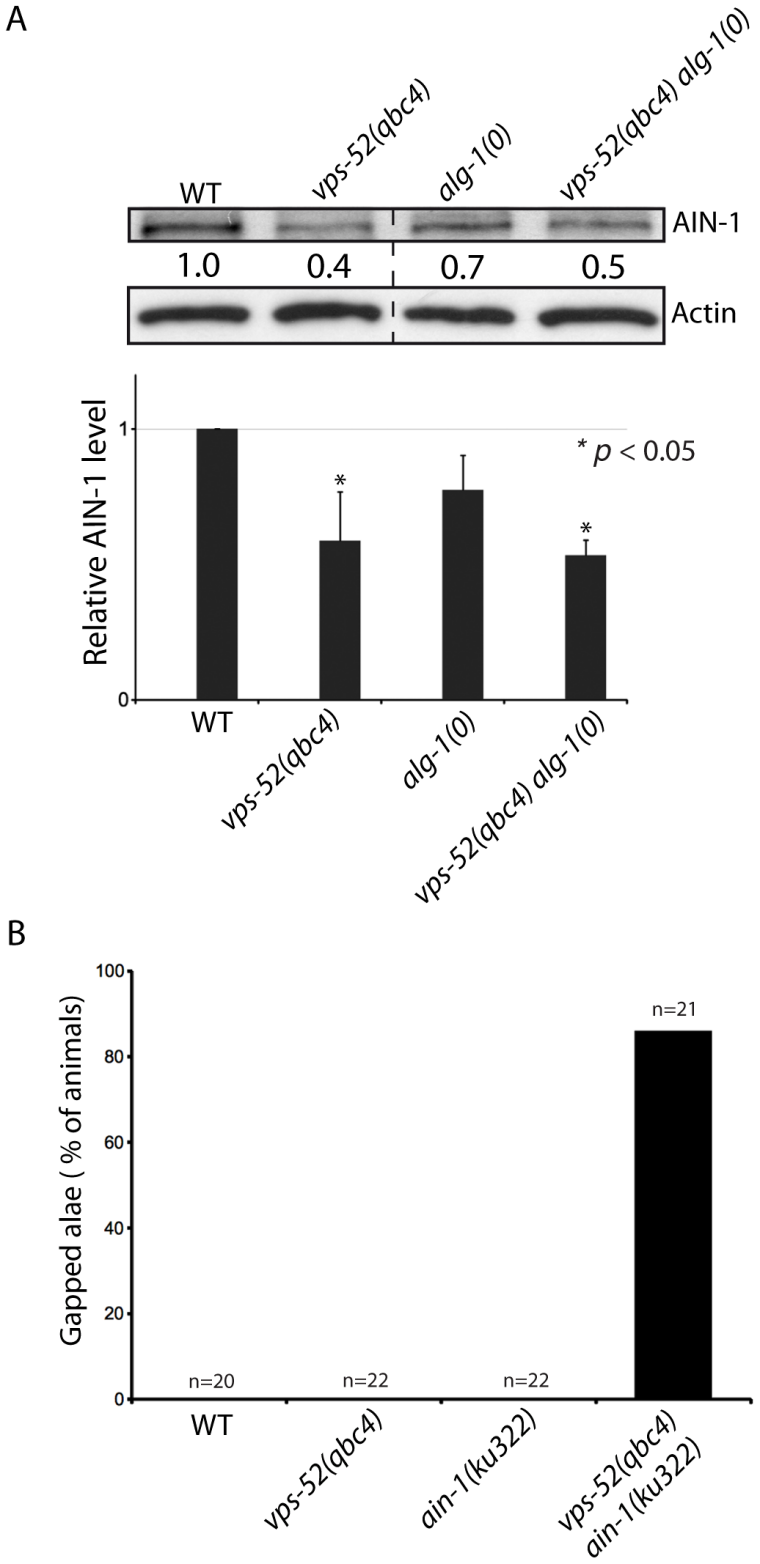
Effects of *vps-52* on the GW182 protein and GW182 mutant animals. **A**) Abundance of the GW182 protein, AIN-1. (Top) Populations of the indicated genotyped were synchronized to the adult stage and Western blot performed on these samples. Actin served as loading control. (Bottom) Quantification of the AIN-1 signal compared with the level found in wild type worms (WT: 1). The error bars represent standard deviation from three independent experiments. *p* values were obtained using a two-sided Student's test. **B**) Synergy between *vps-52* and *ain-1* mutants. The indicated strains were grown at 15°C and the continuity of the cuticular alae analyzed in young adult animals. The number of animals scored (n) is indicated.

In order to gain insights into the steps at which *vps-52* regulate miRNA activity, we investigated the physical association of VPS-52 with components of the microRNA pathway in *C. elegans*. Using immunoprecipitation, we did not observe physical interaction between VPS-52 and the miRISC components AIN-1 or ALG-1 (data not shown). We next investigated if the GARP mutations affected the abundance of key miRNA pathway proteins in *C. elegans*. While we did not detect any effect on the DCR-1 or ALG-1/2 protein levels (Figure S3), the abundance of the GW182 AIN-1 protein was reduced in both *vps-52* and *vps-52 alg-1* mutants (Figure 4A) and this without a corresponding decrease in the expression at the mRNA level of the *ain-1* gene (Figure S3B). In order to determine if this effect of *vps-52* was specific to *ain-1*, we addressed genetically the effect of loss of *vps-52* in *ain-1* function. Interestingly, *vps-52(qbc4)* and the loss-of-function *ain-1(ku322)* mutant synergized, as their combination induced a strong enhancement of seam cell defects (Figure 4B). Thus, *vps-52* may also modulate miRNA activity in parallel to *ain-1*. It is likely that the *ain-2* gene, which encodes a second GW182 protein that provides redundant miRISC function with AIN-1 [13], is also affected by the loss of *vps-52*. We therefore examined the effect of *vps-52* on a AIN-2::GFP translational reporter [13] to overcome the lack of specific antibodies. We observed that the knockdown of *alg-1* in the *vps-52(qbc4)* mutant induced a moderate decrease of AIN-2::GFP abundance notably in vulva cells (Figure S4). The moderate effect detected with this transgenic GW182 reporter may be due to its inability to recapitulate the dynamics and expression level of the endogenous AIN-2 protein. Altogether these results support that the GARP complex may impinge on the abundance of the GW182 protein.

**Figure 5:**
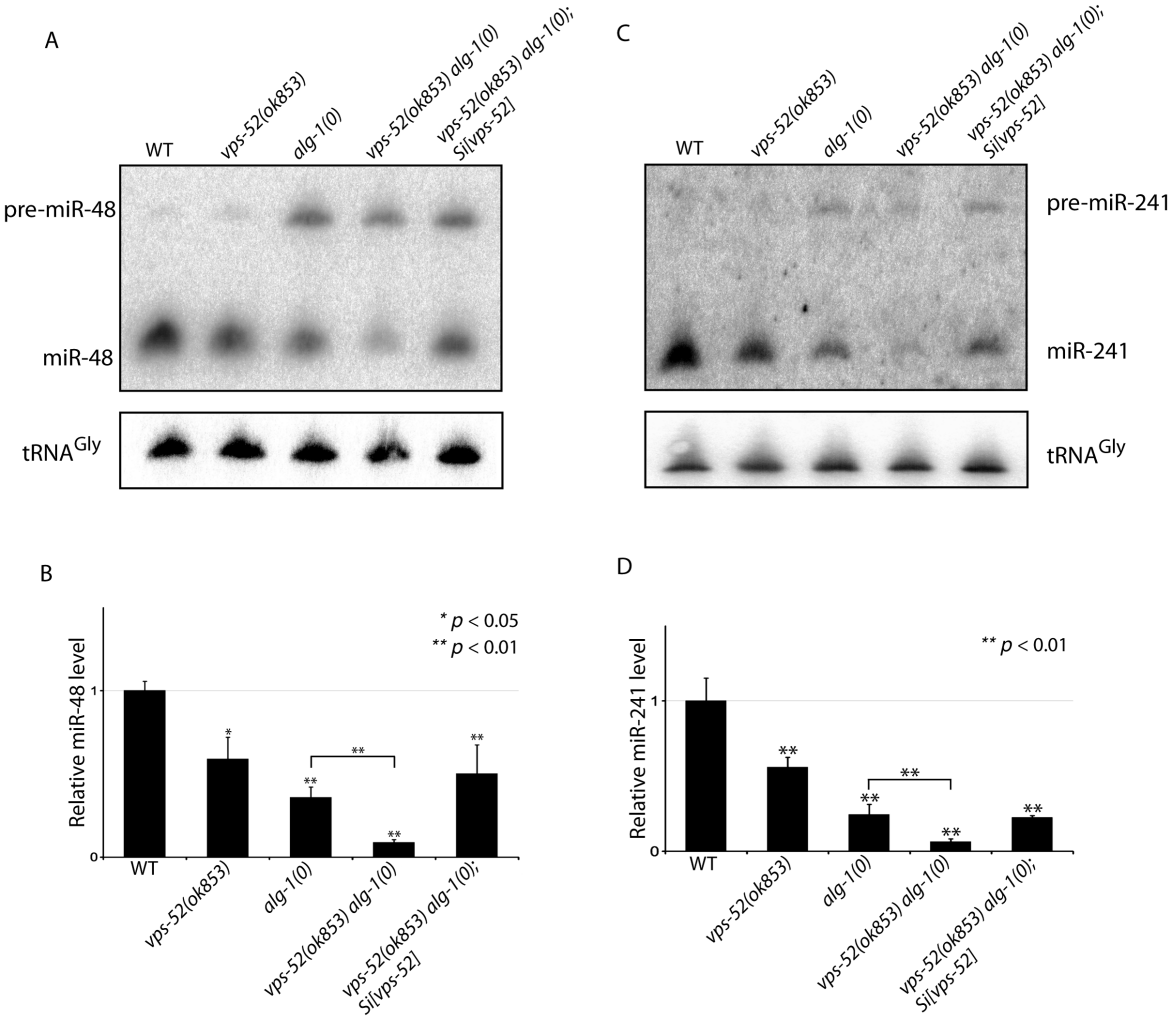
Synergistic effects of *vps-52* on the abundance of mature *let-7* family miRNAs. (**A, C**) The precursor and mature forms of miR-48 (**A**) and miR-241 (**C**) were investigated by Northern blotting in mid-L3 synchronized animals. The tRNA^Gly^ was used as control RNA. (**B, D**) The abundance of the miR-48 (**B**) and miR-241 (**D**). miRNAs level was measured by quantitative real-time PCR (TaqMan assay) in mid-L3 synchronized mutant animals and compared with the level found in wild type worms (WT: 1). The small nucleolar RNAs sn2841 was used as the normalization control. The error bars represent the standard deviation of three independent experiments and *p* values were obtained using a two-sided Student *t*-test.

Next, we determined the effects of the alteration of *vps-52* on miRNA levels. Considering that several of the observed genetic interactions are congruent with reduced activity of the let-7 family miRNAs, we monitored their abundance. While in the single *vps-52* mutant, we observed a significant decrease of the let-7 family miRNA members miR-48 and miR-241, their levels were further reduced in *vps-52 alg-1(0)* double mutant (Figure 5). Moreover, this reduced miR-48 and miR-241 abundance was rescued to the levels found in the single *alg-1(0)* mutant upon expression of a *vps-52* rescue transgene (Figure 5). The level of primary and precursors miRNA molecules remained intact or was mildly altered (Figures S5A and 5), supporting that the decreased miRNA abundance is not likely due to diminished transcription or biogenesis of these miRNAs. The diminished miRNA abundance induced by the loss of *vps-52* was not restricted only to these two let-7 family miRNAs. In *vps-52* mutant, the abundance of three other miRNAs tested was also significantly reduced as observed for miR-48 and miR-241 (Figure S5C). This result supports that the loss of *vps-52* impinges on a broad or general manner on miRNAs. We conclude that the GARP-mediated modulation of miRNA function, observed upon loss of GARP in *alg-1(0)* and multiple other mutants of miRNAs and their targets, is likely underpinned by the lowered abundance of the miRISC component GW182 as well as reduced miRNA abundance.

## Discussion

Employing a genetic screen for *alg-1/2* Argonaute interactors, we have identified the gene *vps-52*, encoding a component of the GARP complex, as a genetic enhancer of the miRNA pathway activity in *C. elegans*. The *vps-52* mutants displayed weakly penetrant miRNA defects, and correspondingly mild molecular alterations of the pathway. However, upon *vps-52* loss, a positive role was uncovered by the induction of increased defects in sensitized mutant backgrounds for Argonautes, miRNAs and their targets. The phenotypes and interactions we observed for *vps-52*, establish it as a modulator of miRNA activity rather than a core pathway component, which is incidentally the type of factors expect to be retrieved from a modifier genetic screen.

We encountered similar phenotypes for the mutants of *vps-52* and *vps-53*, and both synergized with the *alg-1* and *let-7* mutants. Given that this two genes encode components of the GARP complex, it is likely that the observed regulation of miRNA activity corresponds to the impairment of a GARP complex function, rather than other putative additional role(s) of these genes. The GARP complex is involved in the tethering of endosome-derived vesicles reaching the TGN, in a recycling pathway known as ‘retrograde transport’ (reviewed in [46]). This pathway allows the recycling of proteins such as the cation-independent mannose 6-phosphate receptors in mammalian cells, and its impairment in both mammals and yeast leads to protein missorting [22], [47]. Consequently, we hypothesize that the mechanism underlying the observed regulation of miRNA activity is related to the known function of the GARP complex in tethering endosome-derived vesicles at the TGN and the consequences of it [46]. Noteworthy, a different sensitized *let-7* background was previously employed in a genome-wide screen for regulators of the miRNA pathway [37]. Among the top hits identified were components of the COG (Conserved Oligomeric Golgi) complex, a vesicle tethering complex [48], exemplifying again the link between Golgi trafficking functions and miRNA activity.

Numerous studies have now reported on several endomembrane-related aspects of the miRNA pathway in diverse organisms: 1) the presence of Argonaute at the Golgi of certain cultured cells [49], [50]; 2) the co-fractionation of pathway components with Multivesicular Bodies (MVBs) and the negative effects of disrupting components of this compartment [4]; 3) the association of Argonautes and Dicer with the endoplasmic reticulum [51], [52]; 4) the miRNA regulatory effects of the BLOC-3 complex [53]; 5) those of disrupting the isoprenoid producing enzymes of the mevalonate pathway [54], [55] and; 6) the selective autophagic degradation of Dicer and Argonaute [9], [10] and *C. elegans* AIN-1 (GW182) protein [11]. Although all these findings may be underpinned by different mechanisms, they highlight the importance of membrane-regulated aspects on the function of the miRNA pathway. The impairment of autophagy leads to suppression of defective miRNA-mediated gene silencing in *C. elegans* and affects the abundance of AIN-1 [11]. Thus, it is conceivable that lysosomal mediated pathways, such as autophagy, underpin the lessened GW182 abundance we observed upon the loss of GARP.

An interesting possibility regarding the membrane association of the miRNA pathway is that it relates to the sorting and secretion of miRNAs in MVB-derived exosomes. Indeed, circulating miRNAs have been detected in diverse body fluids and proposed to be involved in some form of intercellular communication (reviewed in [56]). Similarly, if miRNA secretion were eventually used to alter gene expression in other cells, it should be expected to alter the cell autonomy of miRNA action to certain extent. In *C. elegans* this aspect has been only studied for *lin-4* in the seam cells, where this miRNA functions cell autonomously [57]. Nonetheless, the sorting of the miRNA and pathway components in exosomes, their putative secretion and role in intercellular communication in *C. elegans* and other organisms are research topics that require further exploration.

An alternative and non-mutually exclusive possibility with that of miRNA secretion, is that the membrane association of the miRNA pathway could be part of a process that facilitates certain transitions occurring during the course of miRNA action [5]. This process being facilitating rather than necessarily required, its absence would only impair, but not completely abolish, miRNA-mediated gene regulation. It can be envisioned that the miRISC components would be associated with endomembranes, such as endosomal vesicles, to facilitate a transition of the miRISC, including, possibly, the recycling of miRISC components, its assembly or disassembly. In this context, impairing the GARP complex function in retrieving endosomal vesicles carrying miRISC components or other factors required for proper miRISC activity would have two foreseeable consequences: i) missorting of miRISC components, such as the GW182 proteins and; ii) a ‘block’ of the miRISC at membranes. As a result, the affected complexes would be disallowed from engaging in further repression of target mRNAs, and also from participating in the accumulation of new miRNAs. Although a role for GW182 in regulating miRNA stability has been recently proposed in mammalian cells [58], other reports indicated that the abundance of miRNAs is not affected by the absence of the GW182 proteins [13], [59], suggesting that miRNA abundance and GW182 accumulation can be uncoupled. Consistently with these last reports, we did not find a decrease in miRNA abundance upon the loss of AIN-1 (Figure S5B–C).

In addition to the processes of miRNA biogenesis and core effector functions, the understanding of subsequent phase(s) of microRNA activity will likely unveil the existence of new components that facilitate miRNA activity and regulate its recycling and turnover. The present trends of discoveries suggest that these facets may be, at least in part, dependent on processes occurring at the interface of endomembranes. Future studies will be required to establish the mechanism by which GARP regulates miRNA activity. Similarly, further research will be helpful to better understand the mechanisms by which membrane-based processes regulate the function of miRNAs.

## Materials and Methods

### Culture conditions and general methods

Worms were cultured in standard conditions [60]. All experiments were performed at 20°C unless otherwise noted. The strains used in the present study were outcrossed four times before analysis (detailed on the strains can be found in Text S1). The RNAi by feeding was performed on nematode growth media (NGM) plates containing 1 mM IPTG (Isopropyl β-D-1-thiogalactopyranoside) after overnight induction (25°C) of the bacterial culture. The *alg-1/2*(RNAi) was performed as described in [15]. The *alg-1*(RNAi) was performed with a construct targeting the *alg-1*-specific N-terminal gene region [35].

### Genetic screen and mutant identification

The genetic screen followed a design to isolate enhancers of the queried gene (including synthetic lethal interactors) previously used in *C. elegans* [21], [61]. EMS was used to mutagenize *alg-2(ok304)* worms carrying an extrachromosomal array containing *GFP*::*alg-2* copies (strain MJS11). From a pool of 1,000 mutagenized fluorescent F1, F2 clones where the non-integrated array becomes necessary for worm survival, evidenced by a homogeneous population of GFP expressing animals were kept and further investigated. Next, single mutant strains (with no transgenic array or *alg-2(ok304)*) were obtained and tested as follows. Upon outcrossing with wild type N2 males, a random set of F2 worms (chosen to be GFP negative, without the *alg-2(ok304)* deletion), was fed with *alg-1/2(RNAi)* and the mendelian segregation of a discernible miRNA-related phenotype (gapped alae or bursting) in the RNAi condition was assessed. One single mutant strain displaying increased defects upon *alg-1/2*(RNAi) with the expected segregation frequency was selected for further characterization.

Using *alg-1/2(RNAi)* the mutant locus was SNP mapped to the X chromosome in the genetic interval (−2.9, −0.76 cM). Mutations inside the interval were unveiled by whole genome sequencing (in collaboration with Dr Don Moerman and the British Columbia Cancer Agency). Only a single nonsense mutation inside the interval (in the F08C6.3 gene) was found. A transgenic strain carrying the wild-type *vps-52* gene, rescued all the visible defects of the mutant strain. To note, the obtained *vps-52(qbc4)* mutant is sensitive to both germline and somatic RNAi (data not shown).

### Plasmids

The Mos transposase plasmid (pJL44) and co-injection markers (pGH8, pCFJ90, pCFJ104) were used following the MosSCI method [62]. To generate the *vps-52* rescue plasmid MSp166 (*Pvps-52::vps-52::mCherry::vps-52* 3′UTR), two genomic regions comprising the whole *vps-52* gene were amplified, introducing a NotI site adjacent to the stop codon and terminal restriction digestion sites (AvrII and BsiWI). Upon NotI ligation, the resulting gene fragment was introduced into double digested (AvrII, BsiWI) pCFJ151 plasmid and verified by sequencing. Using the introduced C-terminal NotI site, a mCherry NotI cassette was ligated to produce MSp166. The oligonucleotides used for plasmid construction are listed in Text S1.

### Transgenic strains

A single copy transgenic line containing the promoter, coding sequence and both 5′ and 3′ untranslated regions of the *vps-52* gene was obtained as follows. Mutant *unc-119(ed9)* worms (strain EG4322) were injected with a mix of the MSp166 plasmid, Mos transposase and marker plasmids following the MosSCI method [62]. The obtained transgenic lines were processed according to the heat-shock protocol of the method. The integrity of the single copy insert was tested by PCR and sequencing. The obtained line (carrying the *qbcSi01* transgene) was then used for crosses.

### Western blotting

Synchronized worm populations of the desired stage were disrupted and dissolved in Laemmli buffer. Protein bands in the immunoblots were visualized using the western lighting plus ECL kit (Perkin-Elmer). Antibodies were used at the following dilutions. Actin-HRP (1∶10,000); rabbit anti-ALG-1 (1∶5,000); rat anti-AIN-1 (1∶10,000) [13]; rabbit anti-ALG-1/2 (1∶2,000) [63]; rabbit anti-DCR-1 (1∶5,000) [64].

### Real-time quantitative PCR

Total RNA from synchronized worm populations was prepared using Tri-Reagent (Sigma-Aldrich). Reverse transcription was performed with the high capacity cDNA reverse transcription kit (Life technologies). A 7900HT PCR system was used for quantitative real-time PCR. SYBR Green I (Invitrogen) was used to monitor pri-miRNA and mRNA levels. TaqMan small RNA assays (Life technologies) were used to measure miRNA levels (miR-48/miR-241, let-7) following the manufacturer protocol. The *sn2841* (small nucleolar RNA) Taqman assay was used as control. The oligonucleotides used for qPCR are listed in Text S1.

### Northern blotting

Total RNA was separated by gel electrophoresis, transferred into a Genescreen plus membrane (Perkin-Elmer) and crosslinked using 1-ethyl-3-(3-dimethylaminopropyl) carbodiimide hydrochloride (EDC) (Sigma) as described in [65]. DNA probes radiolabeled with the Starfire system (IDT) were hybridized to the membrane. After washing, the membrane was exposed to an image plate and scanned with the FLA-5100 phosphoimager. Image quantification was done using the ImageGauge 4.1 (Fujifilm) software. The oligonucleotide probes are listed in Text S1.

## Supporting Information

Figure S1 Analysis of the expression of VPS-52 during animal development. Micrographs of Nomarski and mCherry fluorescence (in red) for L2 larvae (L2) and Adult (Ad) stages. Left panels: VPS-52 is expressed in the cytoplasm of hypodermic cells (seam cells nuclei are indicated by arrowheads). Right panels: VPS-52 is expressed in the vulva and its precursor cells (arrowheads) at the indicated developmental stages. Scale bars measure 20 µm.
(TIF)

Figure S2 Analysis of precocious alae synthesis in the *vps-52* and *hbl-*1 mutants. Representative Nomarski micrographs of *hbl-1(ve18)* and suppressed *hbl-1(ve18) vps-52(qbc4)* animals at the early L4 stage. **Left panels**: The early L4 vulva and gonad developmental stages (arrowheads) are indicated. The vulva lineage is abnormal in *hbl-1(ve18)* mutants [24]. **Right panels**: The corresponding worm cuticules are shown (enlarged in the insets). Precocious alae of *hbl-1(ve18)* are indicated by the dotted lines. Scale bars measure 25 µm.
(TIF)

Figure S3 Effect of loss of *vps-52* on the level of miRNA pathway components. **A**) Abundance of the DCR-1 and ALG-1/2 proteins, determined by western blotting of adult worm samples. Actin level was used as loading control. **B**) The *alg-1* and *ain-1* mRNA levels were measured by quantitative real-time PCR in adult animals and compared with the level found in wild type worms (WT: 1). The *tba-1* mRNA was used as control RNA. The error bars represent standard deviation of three independent experiments.
(TIF)

Figure S4 Analysis of the effects of *vps-52* on AIN-2::GFP fluorescent reporter. **Top panels**: Representative fluorescent micrographs of AIN-2::GFP. Worms populations of indicated genotypes were subjected to control or *alg-1* RNAi by feeding and the GFP fluorescent intensity of L4 animals detected under identical settings (50 ms exposure time). Scale bars measure 20 µm. **Bottom panel**: Quantification of the GFP signal in the vulva cells in Arbitrary Unit (AU) performed with AxioVision 4.8 software (Zeiss). The error bars represent the 95% confidence interval and *p* values were obtained using a two-sided Student *t*-test. The number of animals scored (n) is indicated.
(TIF)

Figure S5 The effect of the *vps-52* and *ain-1* mutants on primary, precursor and mature miRNA abundance. **A**) The primary forms of miR-48 and miR-241 were investigated by real-time quantitative RT-PCR in mid-L3 synchronized animals. The levels of the primary forms were compared with the ones found in wild type worms (WT: 1). The *tba-1* mRNA was used as control RNA. The error bars represent standard deviation of three independent experiments. **B**) Abundance of miR-48 and miR-241 miRNAs in *ain-1* mutant animals. **Left**: Representative Northern blotting of RNA samples from synchronized populations at mid-L3 of wild type N2 (WT) and *ain-1(ku322)* animals. **Right**: The quantification of miRNAs normalized with tRNA^Gly^ (control RNA). The error bars represent the standard deviation from three independent experiments. **C**) Abundance of let-7, miR-1 and lin-4 miRNAs. Representative Northern blotting of RNA samples from synchronized populations at mid-L3 (for lin-4 and miR-1) and at mid-L4 (for let-7) of wild type N2 (WT), *vps-52(ok853)*, *alg-1(0)* and *ain-1(ku322)* animals. For miR-1 Northern, the dashed line indicates that unrelated lanes have been removed between samples. The tRNA^Gly^ was used as control RNA. The quantifications of three independent experiments are shown below each representative Northern. The error bars represent the standard deviation and *p* values were obtained using a two-sided Student *t*-test.
(TIF)

Table S1 Alae defects of the *rab-6.2* and *vps-52* vesicular trafficking mutants.
(DOCX)

Text S1 Description of *C. elegans* strains and oligonucleotides sequences.
(DOC)
