# Supplementary figures and images for: A New Role for the GARP Complex in MicroRNA-Mediated Gene Regulation
Source: PLoS Genet. 2013 Nov 7;9(11):e1003961. doi: 10.1371/journal.pgen.1003961 (PMC3820791; doi:10.1371/journal.pgen.1003961)

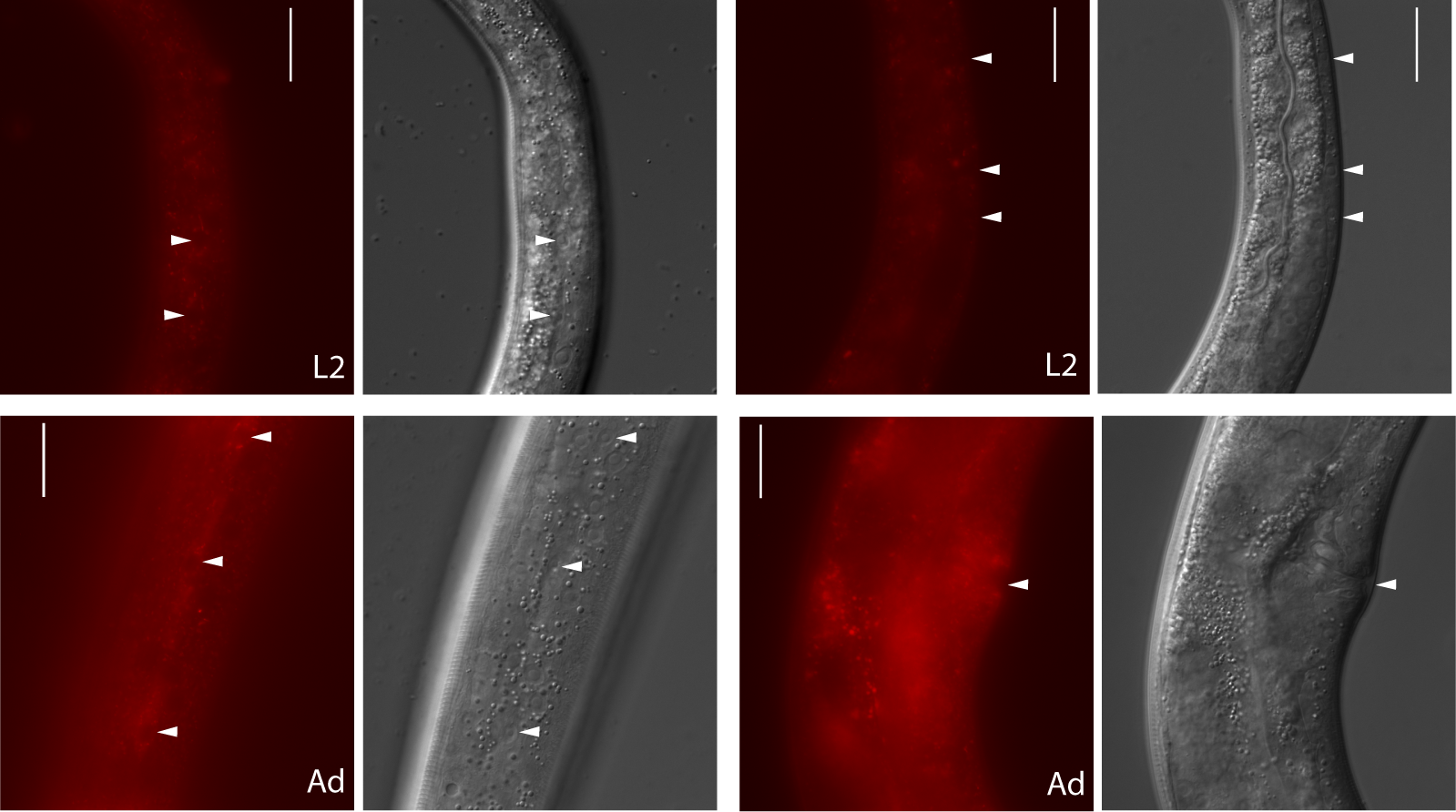

Supplement: Figure S1 — Analysis of the expression of VPS-52 during animal development. Micrographs of Nomarski and mCherry fluorescence (in red) for L2 larvae (L2) and Adult (Ad) stages. Left panels: VPS-52 is expressed in the cytoplasm of hypodermic cells (seam cells nuclei are indicated by arrowheads). Right panels: VPS-52 is expressed in the vulva and its precursor cells (arrowheads) at the indicated developmental stages. Scale bars measure 20 µm. (TIF) [file pgen.1003961.s001.tif]

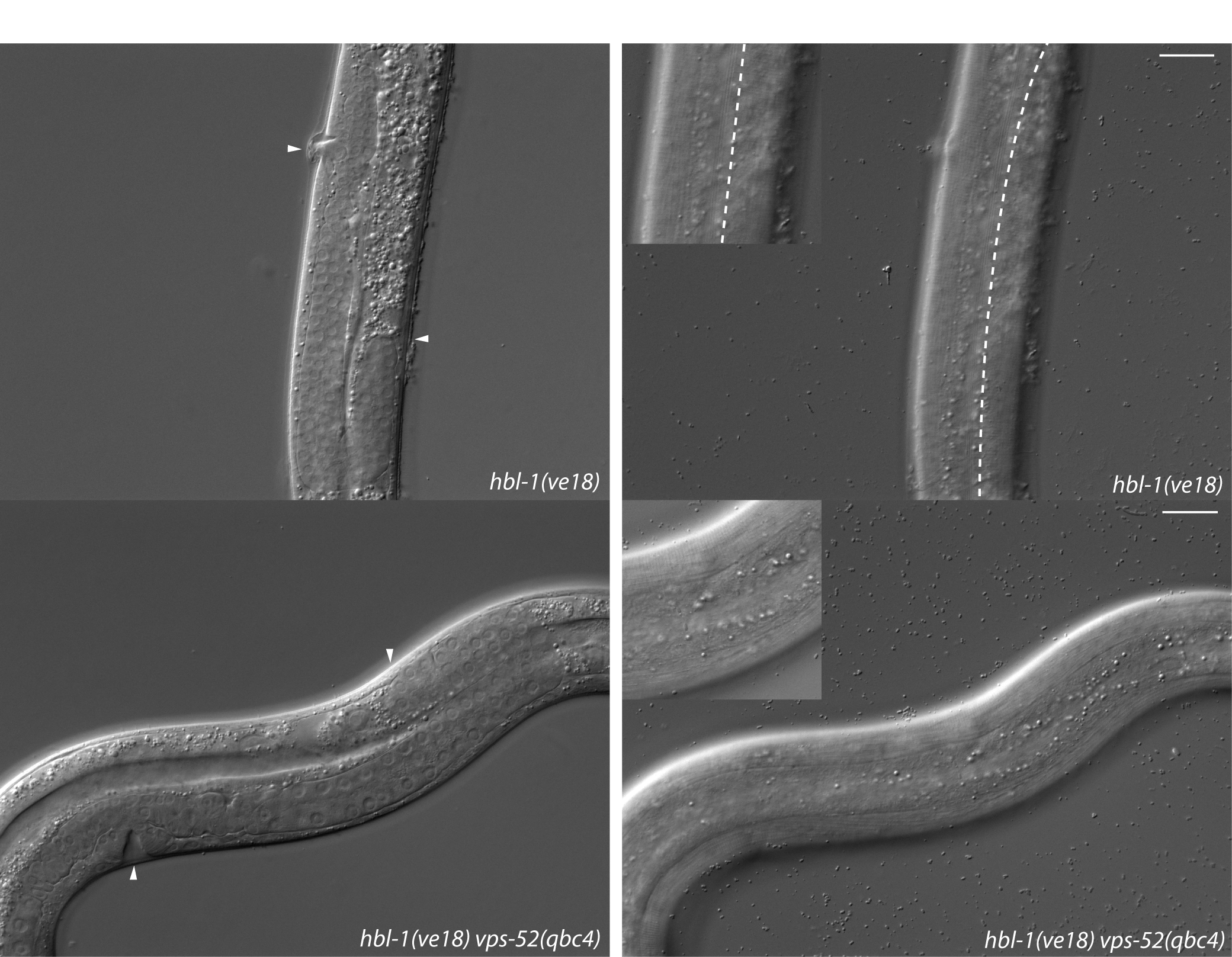

Supplement: Figure S2 — Analysis of precocious alae synthesis in the vps-52 and hbl-1 mutants. Representative Nomarski micrographs of hbl-1(ve18) and suppressed hbl-1(ve18) vps-52(qbc4) animals at the early L4 stage. Left panels: The early L4 vulva and gonad developmental stages (arrowheads) are indicated. The vulva lineage is abnormal in hbl-1(ve18) mutants [24]. Right panels: The corresponding worm cuticules are shown (enlarged in the insets). Precocious alae of hbl-1(ve18) are indicated by the dotted lines. Scale bars measure 25 µm. (TIF) [file pgen.1003961.s002.tif]

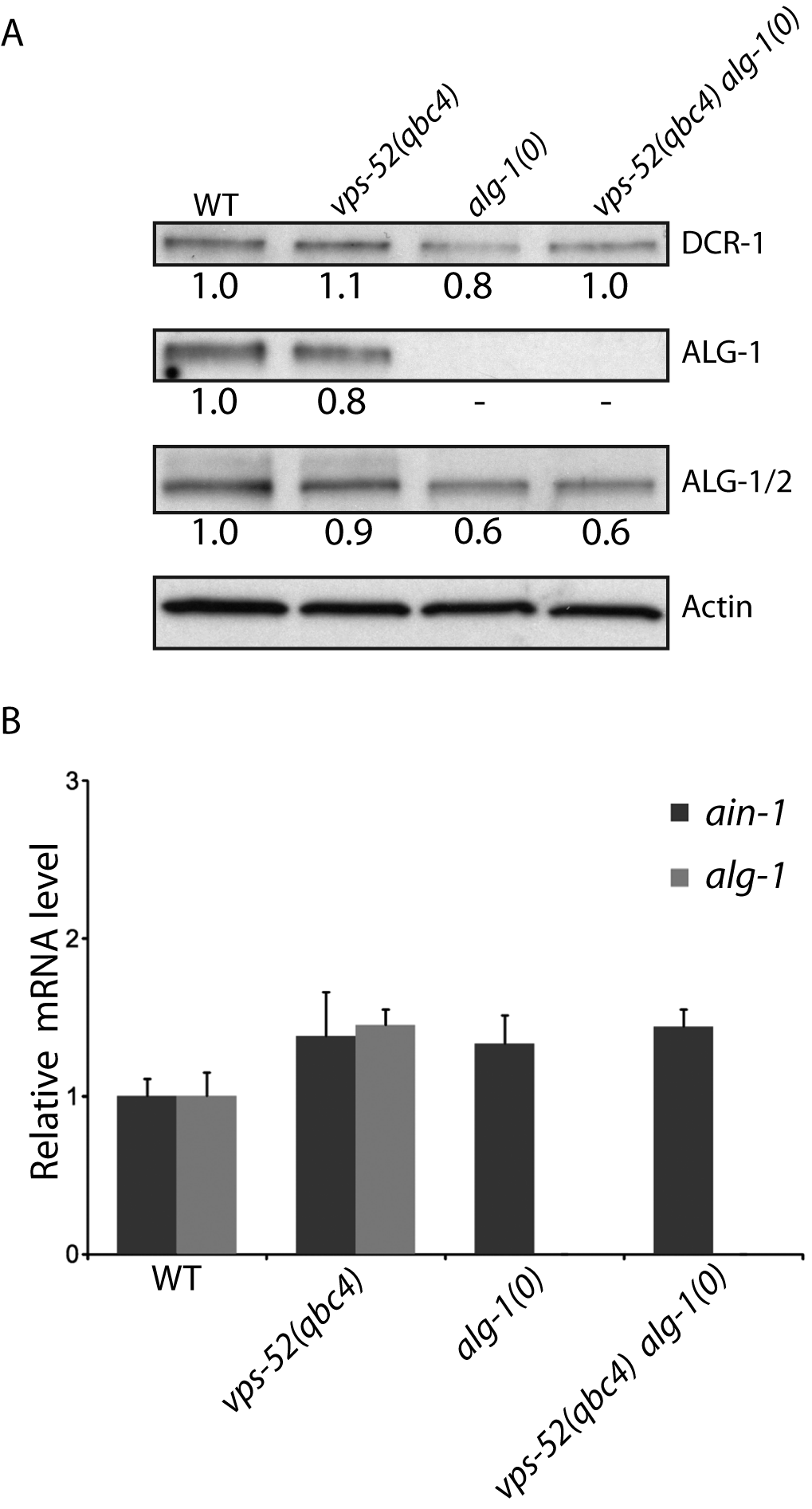

Supplement: Figure S3 — Effect of loss of vps-52 on the level of miRNA pathway components. A) Abundance of the DCR-1 and ALG-1/2 proteins, determined by western blotting of adult worm samples. Actin level was used as loading control. B) The alg-1 and ain-1 mRNA levels were measured by quantitative real-time PCR in adult animals and compared with the level found in wild type worms (WT: 1). The tba-1 mRNA was used as control RNA. The error bars represent standard deviation of three independent experiments. (TIF) [file pgen.1003961.s003.tif]

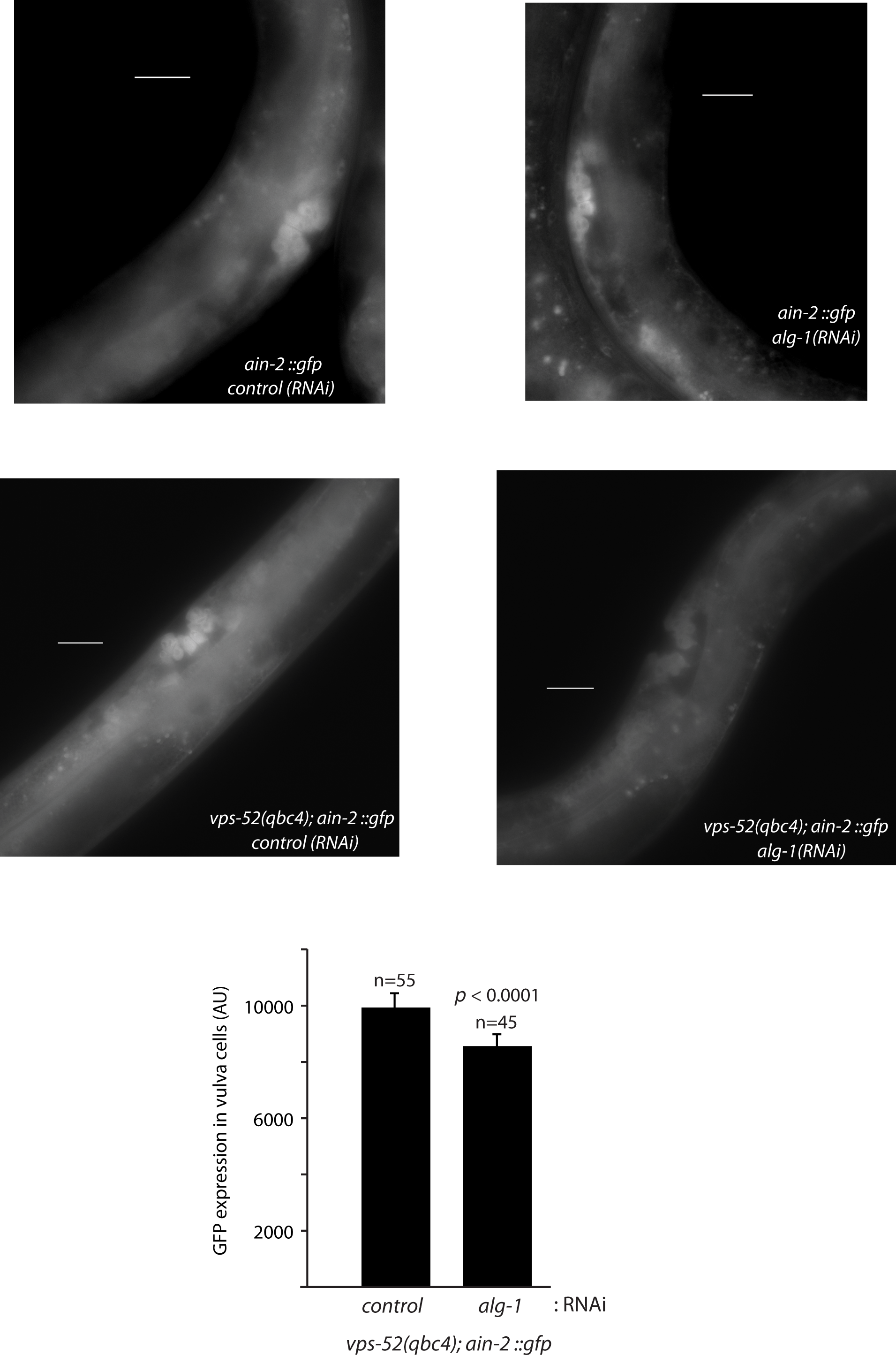

Supplement: Figure S4 — Analysis of the effects of vps-52 on AIN-2::GFP fluorescent reporter. Top panels: Representative fluorescent micrographs of AIN-2::GFP. Worms populations of indicated genotypes were subjected to control or alg-1 RNAi by feeding and the GFP fluorescent intensity of L4 animals detected under identical settings (50 ms exposure time). Scale bars measure 20 µm. Bottom panel: Quantification of the GFP signal in the vulva cells in Arbitrary Unit (AU) performed with AxioVision 4.8 software (Zeiss). The error bars represent the 95% confidence interval and p values were obtained using a two-sided Student t-test. The number of animals scored (n) is indicated. (TIF) [file pgen.1003961.s004.tif]

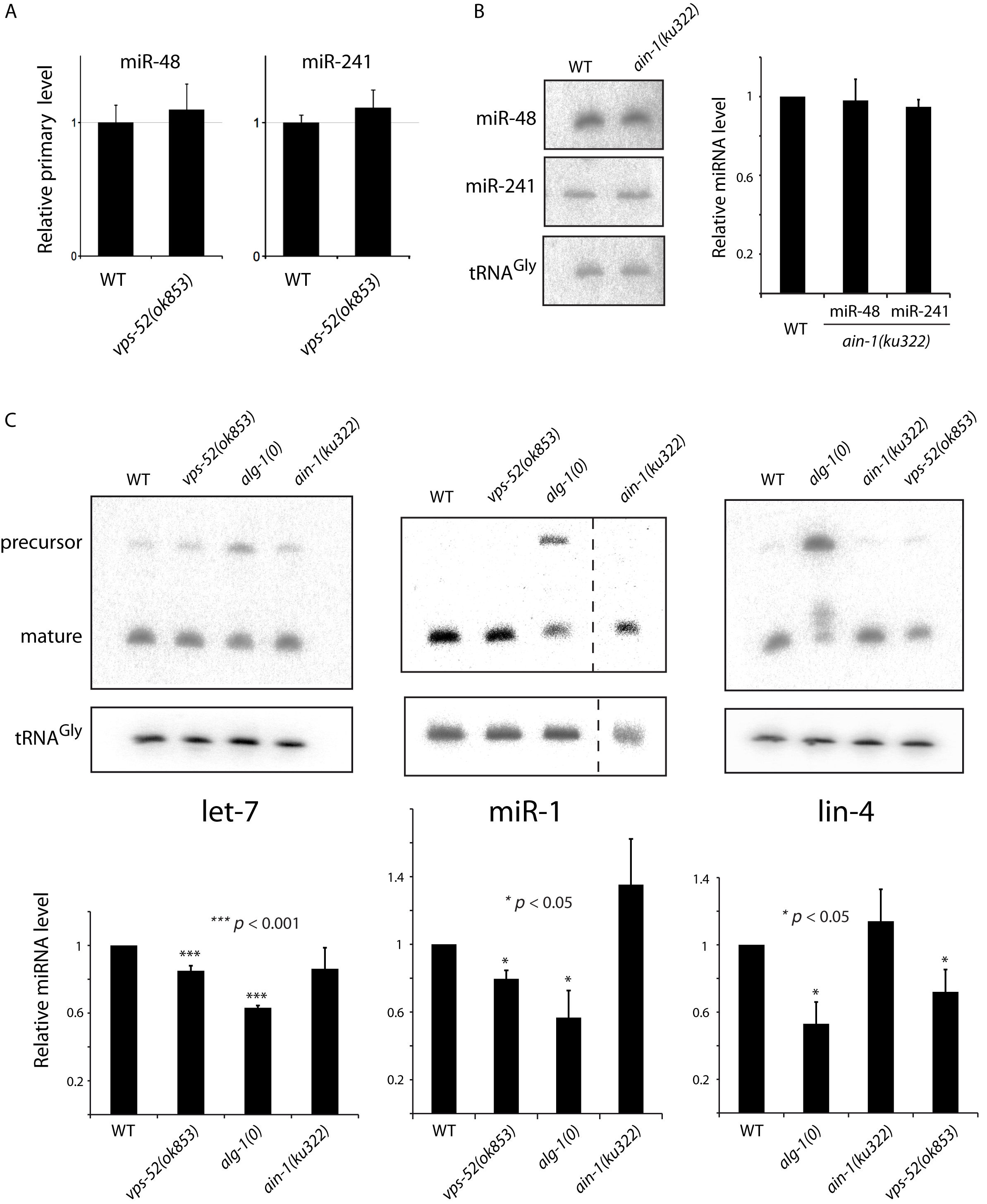

Supplement: Figure S5 — The effect of the vps-52 and ain-1 mutants on primary, precursor and mature miRNA abundance. A) The primary forms of miR-48 and miR-241 were investigated by real-time quantitative RT-PCR in mid-L3 synchronized animals. The levels of the primary forms were compared with the ones found in wild type worms (WT: 1). The tba-1 mRNA was used as control RNA. The error bars represent standard deviation of three independent experiments. B) Abundance of miR-48 and miR-241 miRNAs in ain-1 mutant animals. Left: Representative Northern blotting of RNA samples from synchronized populations at mid-L3 of wild type N2 (WT) and ain-1(ku322) animals. Right: The quantification of miRNAs normalized with tRNAGly (control RNA). The error bars represent the standard deviation from three independent experiments. C) Abundance of let-7, miR-1 and lin-4 miRNAs. Representative Northern blotting of RNA samples from synchronized populations at mid-L3 (for lin-4 and miR-1) and at mid-L4 (for let-7) of wild type N2 (WT), vps-52(ok853), alg-1(0) and ain-1(ku322) animals. For miR-1 Northern, the dashed line indicates that unrelated lanes have been removed between samples. The tRNAGly was used as control RNA. The quantifications of three independent experiments are shown below each representative Northern. The error bars represent the standard deviation and p values were obtained using a two-sided Student t-test. (TIF) [file pgen.1003961.s005.tif]
