# Supplementary material for: A New Role for the GARP Complex in MicroRNA-Mediated Gene Regulation
Source: PLoS Genet. 2013 Nov 7;9(11):e1003961. doi: 10.1371/journal.pgen.1003961 (PMC3820791; doi:10.1371/journal.pgen.1003961)
Supplement: Table S1 — Alae defects of the rab-6.2 and vps-52 vesicular trafficking mutants. (DOCX) [file pgen.1003961.s006.docx]

**Table S1**. Alae defects of the *rab-6.2* and *vps-52* vesicular trafficking mutants.

| Genotype | Adult alae (%) | | |
| --- | --- | --- | --- |
|  | gapped | complete | n |
|  |  |  |  |
| Wild type | 0 | 100 | 30 |
| *rab-6.2(ok2254)* | 0 | 100 | 21 |
| Wild type*; alg-1(RNAi)* | 27 | 73 | 20 |
| *vps-52(qbc4); control(RNAi)* | 5 | 95 | 22 |
| *vps-52(qbc4); alg-1(RNAi)* | 41 | 59 | 20 |
| *rab-6.2(ok2254); control(RNAi)* | 0 | 100 | 21 |
| *rab-6.2(ok2254); alg-1(RNAi)* | 100 | 0 | 20 |

L1 animals of the indicated genotypes were fed with bacteria expressing either control (*control(RNAi)*) or *alg-1* targeting (*alg-1(RNAi)*) dsRNA. The worms were grown at 15° C and scored under Nomarski optics for alae defects (gapped or complete) at young adult stage. The percentage of animals with complete or gapped alae is indicated. The number of animals scored (n) is indicated.
