# Supplementary material for: A New Role for the GARP Complex in MicroRNA-Mediated Gene Regulation
Source: PLoS Genet. 2013 Nov 7;9(11):e1003961. doi: 10.1371/journal.pgen.1003961 (PMC3820791; doi:10.1371/journal.pgen.1003961)
Supplement: Text S1 — Description of C. elegans strains and oligonucleotides sequences. (DOC) [file pgen.1003961.s007.doc]

**A new role for the GARP complex in microRNA-mediated gene regulation**

Alejandro Vasquez-Rifo, Gabriel D. Bossé, Evelyne L. Rondeau, Guillaume Jannot, Alexandra Dallaire and Martin J. Simard

**SUPPORTING INFORMATIONS**

**Strains**

The N2 strain was used as wild type reference. The following strains were used in the present study:

RB574 *alg-2(ok304)* II.

VC446 *alg-1(gk214)* X.

MH2385 *ain-1(ku322)* X.

MJS3 *vps-52(qbc4)* X.

MJS11 *alg-2(ok304); E*x(*sur-5::GFP; GFP::alg-2*)

VC625 *vps-52(ok853)* X.

VC2202 *vps-53(ok2864)* III.

VC2117 *rab-6.2(ok2254)* X.

RG559 *hbl-1(ve18)* X.

OH3646 *lsy-6(ot150)* V; otIs114 [*lim-6*p::GFP + *rol-6(su1006)*] I.

MT2124 *let-60(n1046)* IV*.*

MJS20:

*unc-119(ed3)* III; *vps-52(ok853)* X; qbcSi01 [*pvps-52::vps52::mCherry::vps-52* 3’UTR] II.

MJS21:

*unc-119(ed3)* III; *vps-52(qbc4)* X; qbcSi01 [*pvps-52::vps-52::mCherry::vps-52* 3’UTR] II.

MJS31 *vps-52(qbc4)* X; *vps-53(ok2864)* III.

MJS32 *vps-52(qbc4) alg-1(gk214)* X*.*

MJS33 *vps-52(ok853) alg-1(gk214)* X.

MJS38 *vps-53(ok2864)* III; *alg-1(gk214)* X.

MJS39 *vps-52(ok853) alg-1(gk214)* X; *qbcSi01* II.

MJS40 *vps-52(qbc4)* X; *alg-2(ok304)* II.

MJS41 *vps-52(qbc4) alg-1(gk214)* X; *scm::GFP*

MJS62 *let-7(n2853) vps-52(qbc4)/+* X.

MJS63 *let-7(n2853)* X; *vps-53(ok2864)/+* III.

MJS80 *let-7(n2853) vps-52(qbc4)* X; *qbcSi01* II.

MJS81 *let-7(n2853) vps-52(qbc4)* X; *lin-41(ma104)* I.

MJS85 *vps-52(ok853)* X; *mir-48(n4097)* V.

MJS86 *vps-52(qbc4)* X; *mir-48(n4097)* V.

MJS91 *vps-52(qbc4) hbl-1(ve18)* X.

MJS93 *vps-52(qbc4) ain-1(ku322)* X*.*

MJS95 *vps-52(qbc4)* X; otIs114 I.

MJS96 *vps-52(qbc4)* X; *lsy-6(ot150)* V; otIs114 I.

MJS97 *vps-52(qbc4)* X; *let-60(n1046)/+* IV.

**Oligonucleotides**

The following oligonucleotides were used for plasmids construction:

Amplification first part of *vps-52* gene, incorporates AvrII site

5’-ATAAGAATCCTAGGAGGATCAACTCGAAGCATATTGA-3’

Amplification first part of *vps-52* gene, incorporates NotI site

5’-ATAAGAATGCGGCCGCATATGCTGGTTTGAACCGTTT-3’

Amplification 2^nd^ part of *vps-52* gene, incorporates BsiWI site

5’-ATAAGAATCGTACGTTAACGGTTCTCGAAATATTTTG-3’

Amplification 2^nd^ part of *vps-52* gene, incorporates NotI site

5’-ATAAGAATGCGGCCGCGTAAGAAAAATATTGCCAATGTT-3’

The following oligonucleotides were used for quantitative real-time PCR:

*-ain-1*

Forward: 5’-TTGGATAGTGTGGAGAATGAGC-3’

Reverse: 5’-GATGAGACGATTGTGTTCAAGC-3’

*-alg-1*

Forward: 5’-CAAGTTGTTCCAACATCTGGAG-3’

Reverse: 5’-CGGAGAATTAAATATTTCTTCTAGATCAT-3’

*-tba-1* [1]

Forward: 5’-GTACACTCCACTGATCTCTGCTGACAAG-3’

Reverse: 5’-CTCTGTACAAGAGGCAAACAGCCATG-3’

-pri-*mir-48* [34]

Forward: 5’-TTGGTAGCACCACGTTATTGA-3’

Reverse: 5’-TTGATTCCCCCACAGGTAAG-3’

-pri-*mir-241*

Forward: 5’-GGTAGGTGCGAGAAATGACG-3’

Reverse: 5’-ATTCCGAACCCCTAATCCAAG-3’

The following oligonucleotides were used as probes for Northern blotting:

miR-48 : 5'-TCGCATCTACTGAGCCTACCTCA/Starfire/-3'

miR-241 : 5'-TCATTTCTCGCACTACCTCA /Starfire/-3'

let-7 : 5'-AACTATACAACCTACTACCTCA /Starfire/-3'

lin-4 : 5'-TCACACTTGAGGTCTCAGGGA /Starfire/-3'

miR-1 : 5'-TACATACTTCTTTACATTCCA /Starfire/-3'

tRNA^Gly^ : 5'-GCTTGGAAGGCATCCATGCTGACCATT/Starfire/-3'

**Supplementary Reference**

1. Hoogewijs D, Houthoofd K, Matthijssens F, Vandesompele J, Vanfleteren JR (2008) Selection and validation of a set of reliable reference genes for quantitative sod gene expression analysis in *C. elegans*. BMC molecular biology 9: 9.
